# Distribution of plasma membrane-associated syntaxins 1 through 4 indicates distinct trafficking functions in the synaptic layers of the mouse retina

**DOI:** 10.1186/1471-2202-7-54

**Published:** 2006-07-13

**Authors:** David M Sherry, Robert Mitchell, Kelly M Standifer, Brad du Plessis

**Affiliations:** 1 University of Houston, College of Optometry, Houston, TX 77204, USA; 2 College of Pharmacy, Department of Pharmacological and Pharmaceutical Sciences, University of Houston, Houston, TX 77204, USA

## Abstract

**Background:**

Syntaxins 1 through 4 are SNAP receptor (SNARE) proteins that mediate vesicular trafficking to the plasma membrane. In retina, syntaxins 1 and 3 are expressed at conventional and ribbon synapses, respectively, suggesting that synaptic trafficking functions differ among syntaxin isoforms. To better understand syntaxins in synaptic signaling and trafficking, we further examined the cell- and synapse-specific expression of syntaxins 1 through 4 in the mouse retina by immunolabeling and confocal microscopy.

**Results:**

Each isoform was expressed in the retina and showed a unique distribution in the synaptic layers of the retina, with little or no colocalization of isoforms. Syntaxin 1 was present in amacrine cell bodies and processes and conventional presynaptic terminals in the inner plexiform layer (IPL). Syntaxin 2 was present in amacrine cells and their processes in the IPL, but showed little colocalization with syntaxin 1 or other presynaptic markers. Syntaxin 3 was found in glutamatergic photoreceptor and bipolar cell ribbon synapses, but was absent from putative conventional glutamatergic amacrine cell synapses. Syntaxin 4 was localized to horizontal cell processes in the ribbon synaptic complexes of photoreceptor terminals and in puncta in the IPL that contacted dopaminergic and CD15-positive amacrine cells. Syntaxins 2 and 4 often were apposed to synaptic active zones labeled for bassoon.

**Conclusion:**

These results indicate that each syntaxin isoform has unique, non-redundant functions in synaptic signaling and trafficking. Syntaxins 1 and 3 mediate presynaptic transmitter release from conventional and ribbon synapses, respectively. Syntaxins 2 and 4 are not presynaptic and likely mediate post-synaptic trafficking.

## Background

Syntaxins comprise a large family of membrane-associated proteins that play a critical role in vesicular trafficking and exocytosis. Syntaxins associate with members of the SNAP-25 protein family on the target membrane and with members of the VAMP/synaptobrevin family located in the vesicular membrane to form the SNAP receptor (SNARE) core complex that serves to dock the vesicle to the target membrane and acts as a scaffold for the recruitment of other proteins needed for fusion of the vesicular and target membranes [for review see refs [1,2]]. A large number of syntaxin isoforms have been identified and are associated with specific target membranes and organelles within the cell, such as endoplasmic reticulum, Golgi apparatus, trans-Golgi network, endosomes, and plasma membrane, and are thought to contribute to the specificity of vesicular trafficking between different organelles and subcellular compartments [[3-5]; reviewed in [1,6]]. Syntaxins 1 through 4 specifically associate with the plasma membrane and regulate vesicular trafficking for exocytosis or for insertion of proteins into the plasma membrane [[4,7-10]; reviewed in [1,6]].

A critical role for syntaxins and the other SNARE proteins in nervous tissue is to mediate the fusion of synaptic vesicles to the presynaptic plasma membrane for neurotransmitter exocytosis. Syntaxin 1 is the best-studied of the syntaxins, and is the principal isoform associated with synapses in the brain [[3,4], reviewed in [1,6]]. Syntaxin 1 has been localized ultrastructurally to asymmetric type 1 presynaptic terminals in the brain, but is typically absent from symmetric type 2 synapses [11], suggesting that more than one syntaxin isoform may be involved in synaptic trafficking. Syntaxins 2 through 4 also are expressed in brain [4] but their functions remain unclear. These isoforms are best known for roles in non-synaptic exocytosis and trafficking to the plasma membrane elsewhere in the body [[7-10]; reviewed in [1,6]].

In the retina, both syntaxin 1 and syntaxin 3 are present in presynaptic terminals and are differentially distributed among functionally distinct synapses [12]. Syntaxin 1 is expressed at conventional amacrine cell synapses with transient release characteristics. Syntaxin 3 is found in the glutamatergic ribbon synapses of photoreceptors and bipolar cells, which support very rapid and sustained release, likely via compound fusion of synaptic vesicles [13-15]. Thus, synaptic transmission in the retina is not strictly dependent on syntaxin 1, but also can be mediated by other plasma membrane-associated syntaxin isoforms. Furthermore, the synapse-specific distribution of syntaxins 1 and 3 suggests that the syntaxin isoform present in a terminal may help to shape a synapse's functional attributes. Whether syntaxins 2 and 4 mediate synaptic trafficking in the retina or elsewhere in the central nervous system is unknown.

Many presynaptic proteins associated with neurotransmitter release, and their isoforms, are differentially distributed among synapses. The synapse-specific distribution of many of these proteins is especially well characterized in the retina [e.g., [12,16-23]]. This differential distribution of presynaptic proteins is thought to fine tune the characteristics of neurotransmitter release from the presynaptic terminal. Thus, understanding patterns of presynaptic protein expression at different types of synapses can provide insight into functional differences among different types of synapses. These synapse-specific expression patterns also can serve as a functionally relevant anatomical tool for the identification of specific synapses and circuits.

To better understand the potential functional roles of the syntaxins associated with trafficking to the plasma membrane in the synaptic circuits of the retina, we have examined the cell and synapse-specific distribution of syntaxins 1–4 in the mouse retina using single- and double-labeling immunohistochemistry at the conventional light and confocal microscopic levels. Each syntaxin isoform shows a unique distribution in the synaptic layers of the retina, with little overlap of isoforms, suggesting that each syntaxin isoform has unique trafficking functions.

## Results

### Syntaxins 1 through 4 are expressed in the mouse retina

**Figure 1:**
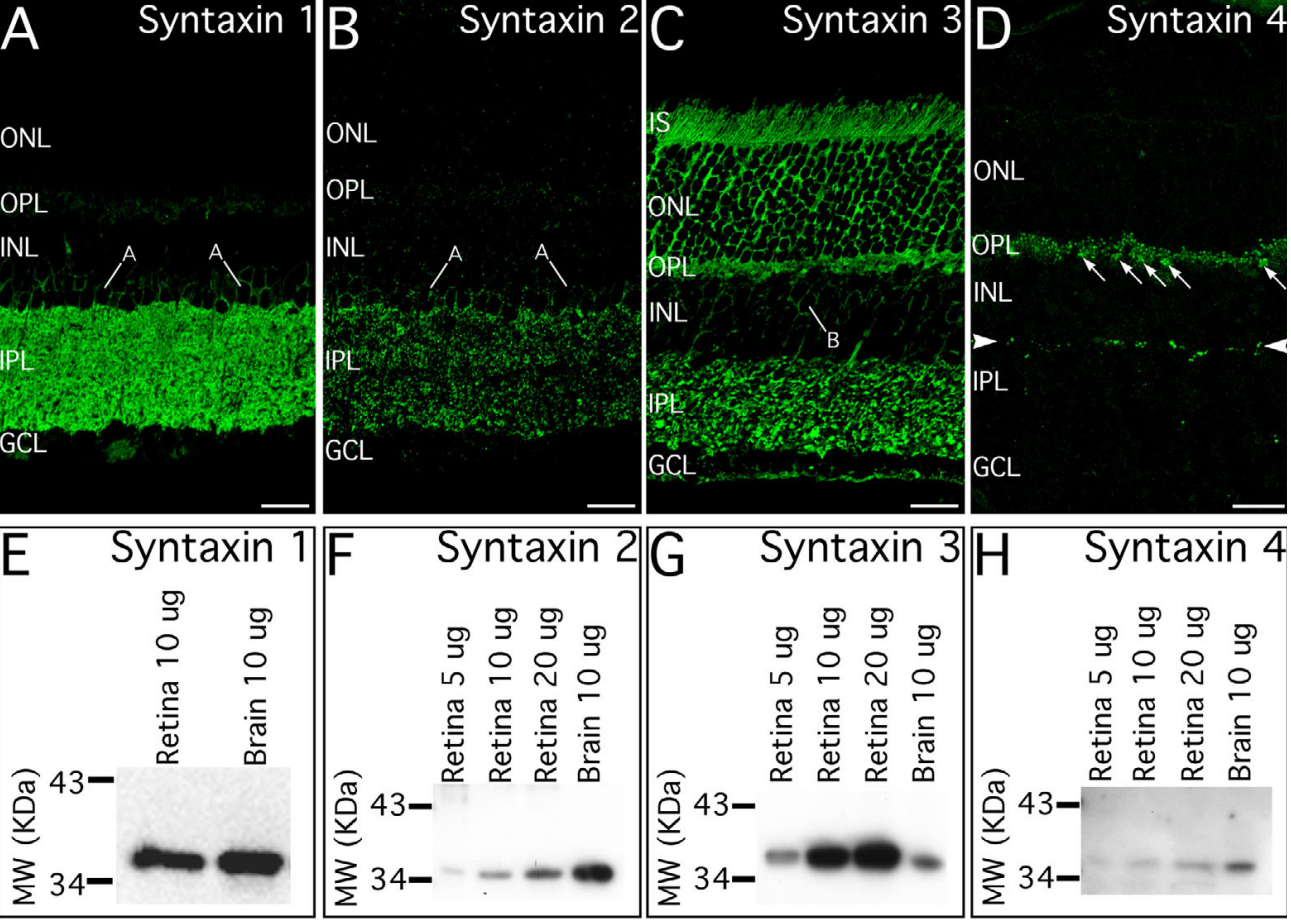
**Syntaxins 1 through 4 are differentially distributed in the synaptic layers of the retina. A**: Syntaxin 1 is localized to amacrine cell bodies (A) and processes in the inner plexiform layer (IPL). **B**: Syntaxin 2 also is localized to amacrine cell bodies (A), but shows a more restricted, punctate distribution in the IPL. **C**: Syntaxin 3 is localized to the glutamatergic ribbon synapses of photoreceptor and bipolar cell terminals in the outer plexiform layer (OPL) and IPL, respectively. Labeling is also present in the cell bodies of the photoreceptors and their inner segments (IS) and in bipolar cell bodies (B) and axons. **D**: Syntaxin 4 shows a very restricted, punctate distribution in the OPL and IPL. In the OPL, clusters of syntaxin 4 processes are associated with cone terminals (arrows). In the IPL, a distinct lamina of syntaxin 4 positive puncta is present at the INL/IPL border (arrowheads), with other puncta sparsely distributed deeper in the IPL (circle). **E–H: **Immunoblots for syntaxins 1 through 4 reveal a band at the appropriate molecular mass in membrane homogenates prepared from mouse retina and brain, confirming the expression of all four syntaxin isoforms in the mouse retina and the specificity of the antibodies and antisera. ONL, outer nuclear layer; INL, inner nuclear layer, GCL, ganglion cell layer. Scale bars = 20 μm for A–D.

Immunoblotting of retinal and brain membrane homogenates showed that each anti-syntaxin antibody recognized a single protein band of the appropriate size (Fig. 1), indicating that each of the four syntaxin isoforms associated with vesicular trafficking to the plasma membrane was present in the mouse retina and brain. Although syntaxins 1 through 4 were all expressed in retina and in brain, relative expression levels differed between brain and retina in an isoform-specific manner. Retina and brain showed roughly similar expression of syntaxin 1, retina expressed more syntaxin 3 than brain, and brain expressed more syntaxin 2 and syntaxin 4 than retina.

Each syntaxin isoform showed a unique distribution in the retina and was localized primarily to the synaptic layers (Fig. 1). Syntaxin 1 labeling was widely distributed in the inner plexiform layer (IPL) and in amacrine cell bodies, but was present only in very few terminals in the outer plexiform layer (OPL). A prominent characteristic of syntaxin 1 labeling was its presence in discrete puncta embedded in a background of diffuse labeling. Syntaxin 2 labeling also was widely distributed in the IPL and in the cell bodies of amacrine cells, but was more punctate in appearance than labeling for syntaxin 1. There was little labeling for syntaxin 2 in the OPL, similar to syntaxin 1. Labeling for syntaxin 3 was present in the cell bodies, inner segments and synaptic terminals of photoreceptors, in bipolar cell bodies and axons, and numerous terminals in the IPL. Syntaxin 4 labeling was the most restricted of all the isoforms and was present in numerous puncta in the OPL, a distinct stratum of puncta at the distal edge of the IPL and in other puncta scattered deeper in the IPL. Syntaxin 4 labeled puncta also were observed in the inner nuclear layer (INL) and some blood vessels (see below).

The isoform-specific differences in expression level and localization suggest that each syntaxin isoform has unique trafficking functions in the retina. To better understand the distribution and potential functional roles of the various syntaxin isoforms in the retina, we performed double-labeling for each syntaxin isoform in combination with a variety of cell and synapse-specific markers with known distribution in the retina.

### Syntaxin 1 and 3 are presynaptic and differentially expressed between conventional and ribbon synapses

Syntaxin 1 and 3 isoforms have been examined in retina previously [12]. Syntaxin 1 has been localized to the conventional synapses of amacrine cells, while syntaxin 3 has been localized to glutamatergic ribbon synapses of photoreceptors and bipolar cells [12].

**Figure 2:**
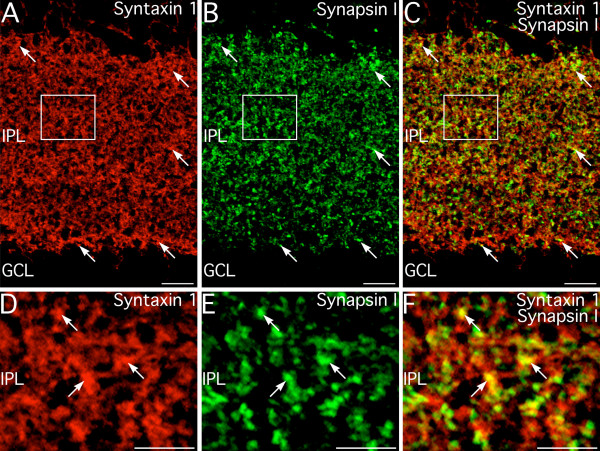
**Syntaxin 1 is associated with conventional synapses in the inner plexiform layer (IPL). A–C: **Confocal microscopy of double labeling for syntaxin 1 and synapsin I, a marker for conventional synapses made by amacrine cells. There is appreciable colocalization of labeling in conventional synaptic terminals (arrows). However, syntaxin 1 labeling is not restricted the presynaptic terminal and is often seen independent of synapsin I labeling. Box indicates area shown in panels D–F. **D–F: **Examination of syntaxin 1 and synapsin I double labeling from the area indicated by the box in panels A–C confirms the localization of syntaxin 1 to conventional synapses. GCL, ganglion cell layer. Images shown from a projection of 12 optical sections with total thickness of 1.6 μm. Scale bars = 10 μm for A–C; 5 μm for D–F.

Double labeling for syntaxin 1 and synapsin I, a marker specific for conventional synapses made by amacrine cells [19], confirmed the localization of syntaxin 1 to amacrine cells and their processes and synapses in the IPL. Labeling for syntaxin 1 and synapsin I colocalized extensively (Fig. 2), confirming the presence of syntaxin 1 at conventional amacrine cell synapses. A substantial portion of syntaxin 1 labeling was diffusely distributed in the IPL and did not correspond precisely to synapsin I labeled puncta, suggesting that syntaxin 1 labeling was not restricted only to the synaptic active zone.

Previous results indicate that syntaxin 3 is associated with the glutamatergic ribbon synapses of photoreceptors and bipolar cells [12]. However, it is not known whether syntaxin 3 is present in the synapses of a recently identified "glutamatergic" amacrine cell that expresses vesicular glutamate transporter 3 (VGLUT3) [24-26], which would imply a functional association of syntaxin 3 with glutamatergic transmission rather than with ribbon synapses exclusively. To test this, we examined colocalization of syntaxin 3 with several markers specific for conventional synapses, glutamatergic ribbon synapses, and VGLUT3.

**Figure 3:**
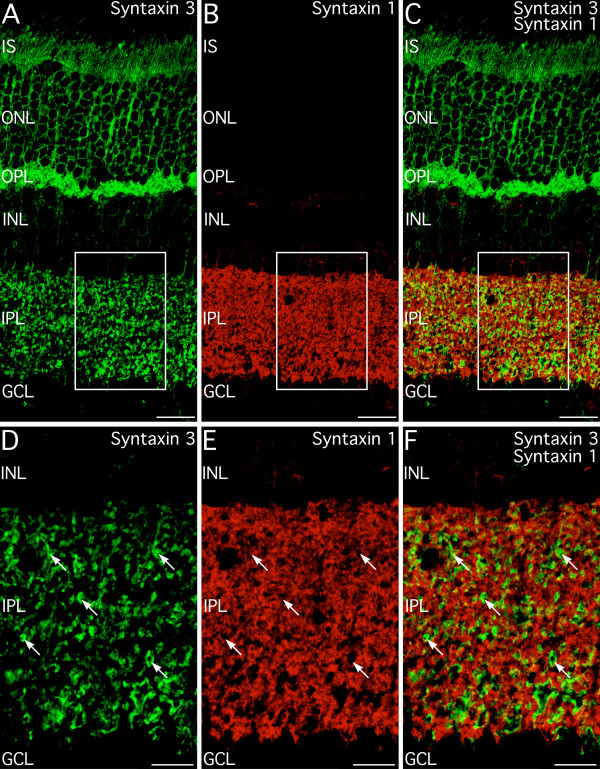
**Syntaxin 3 does not colocalize with syntaxin 1. A–C: **Syntaxin 3 is localized to the glutamatergic photoreceptor terminals in the outer plexiform layer (OPL) and numerous bipolar cell terminals in the inner plexiform layer (IPL). Labeling is also present in the cell bodies and inner segments of the photoreceptors and in bipolar cell bodies and axons. Syntaxin 1 labeling is restricted to amacrine cells and amacrine cell processes. Images shown from a projection of 10 optical sections with total thickness of 1.93 μm. Box indicates area shown in panels D–F. **D–F: **Examination of syntaxin 3 and syntaxin 1 immunolabeling at higher magnification confirms that these syntaxin isoforms are present in different sets of synaptic terminals. Arrows indicate bipolar cell terminals labeled for syntaxin 3. Images shown from a projection of 15 optical sections with total thickness of 2.04 μm. IS, inner segments; ONL, Outer nuclear layer; INL, inner nuclear layer, GCL, ganglion cell layer. Scale bars = 20 μm for A–C; 10 μm for D–F.

**Figure 4:**
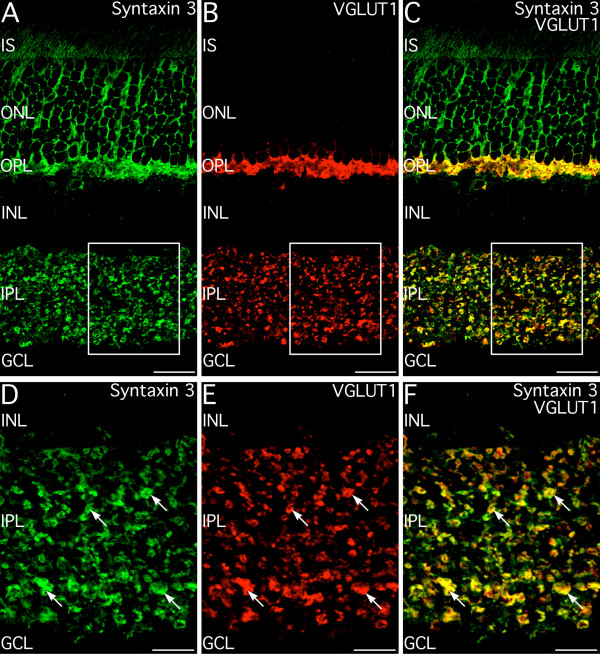
**Syntaxin 3 is associated with the glutamatergic ribbon synapses of photoreceptors and bipolar cells**. **A–C: **Labeling for syntaxin 3 and VGLUT1, a marker for ribbon synapses, colocalize extensively in the photoreceptor terminals in the OPL and in bipolar cell terminals in the IPL. Box indicates area shown in panels D–F. **D–F: **Examination of syntaxin 3 and VGLUT1 immunolabeling in the IPL at higher magnification confirms that all bipolar cell terminals contain syntaxin 3. Images shown are from a single optical section. IS, inner segments; ONL, Outer nuclear layer; INL, inner nuclear layer, GCL, ganglion cell layer. Scale bars = 20 μm for A–C; 10 μm for D–F.

Double labeling for syntaxin 3 and syntaxin 1 directly demonstrated that syntaxin 1 and 3 were restricted to different terminals, although there often was close apposition of syntaxin 3- and syntaxin 1-containing terminals (Fig. 3). Double labeling for syntaxin 3 and the conventional synapse marker synapsin I produced similar results (not shown) indicating that syntaxin 3 was absent from most conventional synapses. Double labeling for syntaxin 3 and vesicular glutamate transporter 1 (VGLUT1), which is expressed at all photoreceptor and bipolar cell ribbon synapses [27,28], confirmed directly that syntaxin 3 was expressed at the glutamatergic ribbon synapses of photoreceptors and bipolar cells (Fig. 4). All photoreceptor and bipolar cell terminals showed double labeling for VGLUT1 and syntaxin 3, indicating that syntaxin 3 was, in fact, expressed in all ribbon synaptic terminals. Syntaxin 3 labeling, however, was distributed more broadly than VGLUT1 labeling, suggesting that syntaxin 3 was not strictly confined to the active zone.

**Figure 5:**
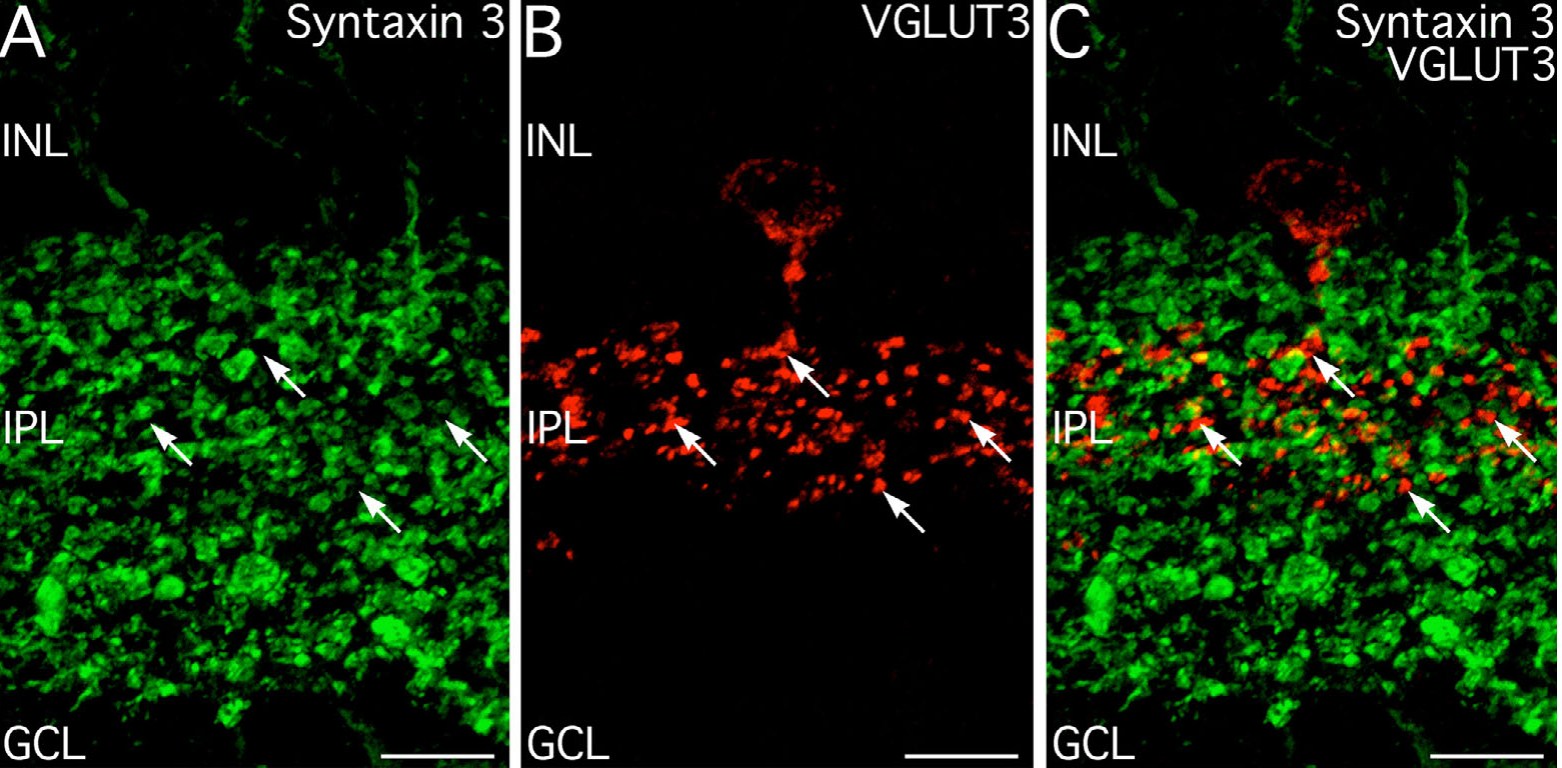
**Syntaxin 3 is not expressed at putative conventional glutamatergic synapses**. **A**. Syntaxin 3 labeling is present in numerous terminals in the inner plexiform layer (IPL), corresponding to the ribbon synapse-containing terminals of bipolar cells (see text for details). **B. **Vesicular glutamate transporter 3 (VGLUT3) is expressed in a putative glutamatergic amacrine cell type and its terminals (arrows) in the mid-IPL. **C**. There is no colocalization of syntaxin 3- and VGLUT3-positive terminals, although there is some contact between these terminals. Images shown from a projection of 14 optical sections with total thickness of 1.89 μm. INL, inner nuclear layer, GCL, ganglion cell layer. Scale bars = 10 μm.

The recent discovery of a small population of putative glutamatergic amacrine cells expressing VGLUT3 [24-26], raised the issue of whether syntaxin 3 also might be expressed at conventional glutamatergic synapses in the retina in addition to ribbon synapses. Double labeling showed that syntaxin 3 was not expressed at conventional synapses of VGLUT3 amacrine cells, although close apposition of some VGLUT3-positive and syntaxin 3-positive terminals was observed (Fig. 5). This result confirmed that syntaxin 3 was associated specifically with the release machinery of ribbon synapses and not with glutamatergic transmission in the retina in general.

### Syntaxin 2 is expressed specifically by amacrine cells

**Figure 6:**
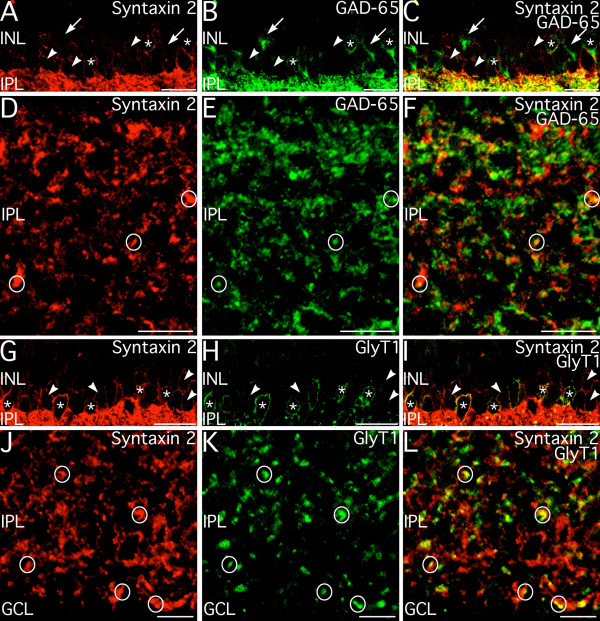
**Syntaxin 2 is expressed in GABAergic and glycinergic amacrine cells. A–C: **Labeling for syntaxin 2 colocalizes with labeling for GAD-65 in numerous GABAergic amacrine cell bodies (*) in the inner nuclear layer (INL). However, syntaxin 2 labeling is not present in all GAD-65-positive amacrine cells (arrows) and many syntaxin 2-positive amacrine cell bodies do not show labeling for GAD-65 (arrowheads). Images shown from a projection of 12 optical sections with total thickness of 2.78 μm. **D–F. **At high magnification, labeling for syntaxin 2 in the inner plexiform layer (IPL) shows only limited colocalization with GAD-65 positive presynaptic terminals (circles). Images shown from a projection of 13 optical sections with total thickness of 1.75 μm. **G–I: **Glycinergic amacrine cells in the INL, labeled for GlyT1, show labeling for syntaxin 2 (*). However, many syntaxin 2-positive amacrine cell bodies are present that do not show labeling for GlyT1 (arrowheads). Images shown from a single optical plane. **J–L: **At high magnification, syntaxin 2 shows colocalization with GlyT1-positive amacrine cell processes in the IPL (circles) although colocalization is imperfect. Images shown from a projection of 6 optical sections with total thickness of 0.73 μm. GCL; ganglion cell layer. Scale bars= 20 μm for A–C and G–I; 10 μm for D–F; 5 μm for J–L.

Syntaxin 2 labeling was found in amacrine cell bodies and numerous puncta in the IPL and was grossly similar to the distribution of syntaxin 1 labeling (see Fig. 1), suggesting that syntaxin 2 was expressed by amacrine cells. Amacrine cells are typically inhibitory, with about half of the amacrine cell population being GABAergic and about half being glycinergic [15,29]. To assess whether syntaxin 2 might be associated specifically with either GABAergic or glycinergic neurotransmission, retinal sections were double labeled for syntaxin 2 and either the 65 kDa isoform of glutamic acid decarboxylase (GAD-65) or glycine transporter 1 (GlyT1) to identify GABAergic and glycinergic amacrine cells, respectively (Fig. 6). Syntaxin 2 labeling colocalized extensively with labeling for GAD-65 and GlyT1 in amacrine cell bodies, indicating that both GABAergic and glycinergic amacrine cell types expressed syntaxin 2 (Fig. 6A–C and 6G–I). Within the IPL, where the GABAergic and glycinergic synapses of the amacrine cells reside, there was scant colocalization of labeling for syntaxin 2 and GAD-65, which labels the presynaptic terminals of most GABAergic amacrine cells (Fig. 6D–F). Similarly, syntaxin 2 labeling showed only limited colocalization with GlyT1 labeling (Fig. 6J–L), although colocalization of syntaxin 2 and GlyT1 labeling was seen more frequently than colocalization of syntaxin 2 and GAD-65.

### Syntaxin 2 shows little colocalization with syntaxin 1 and does not appear to be presynaptic

**Figure 7:**
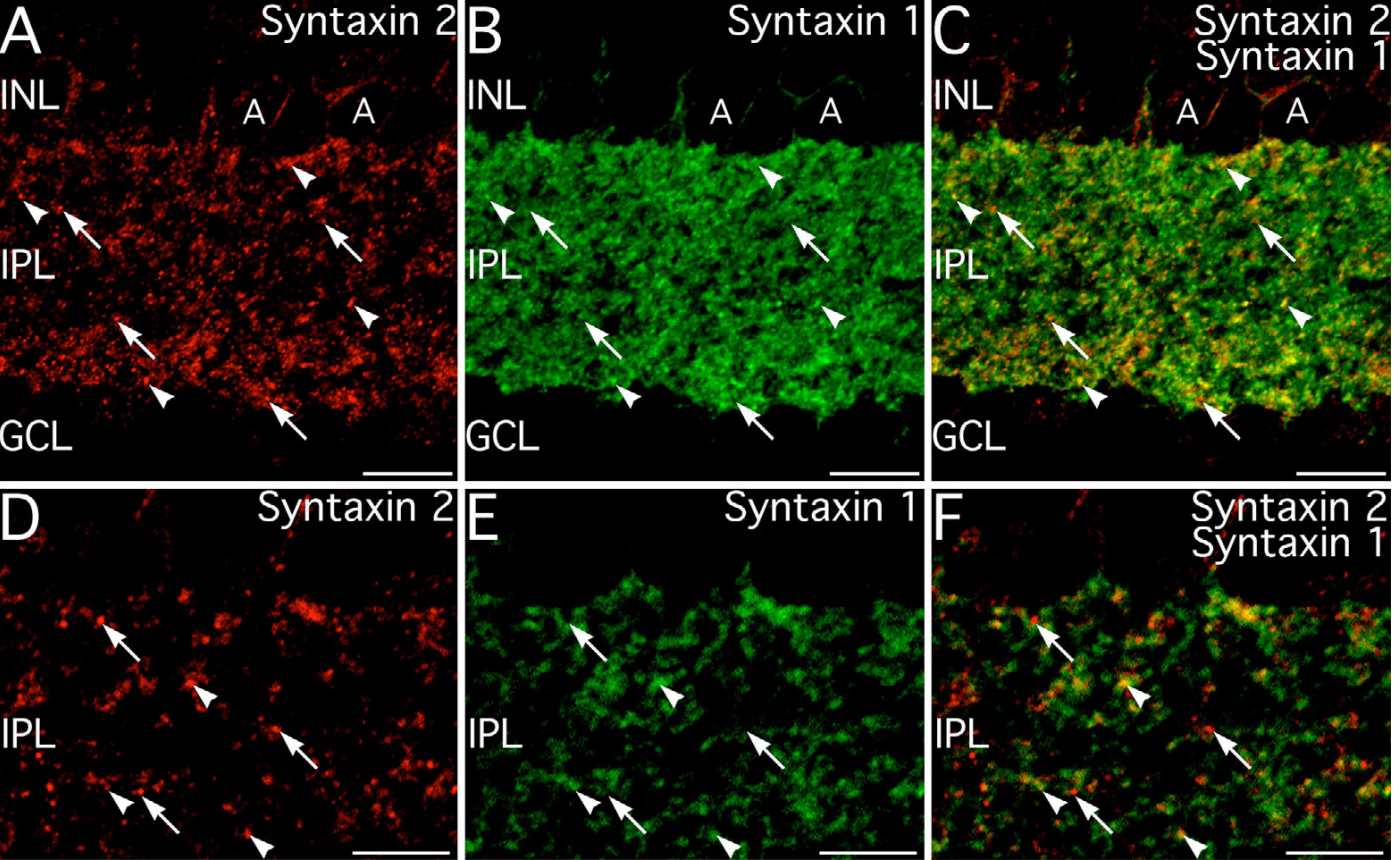
**Syntaxin 2 shows little colocalization with syntaxin I. A–C: **Syntaxin 2 labeling is present in numerous amacrine cells (A) and small puncta throughout the inner plexiform layer (IPL; arrows and arrowheads). Syntaxin 1 is widely expressed in amacrine cell bodies, processes and presynaptic terminals. At low magnification, there is some apparent colocalization of these two syntaxin isoforms in conventional terminals (arrowheads), although many syntaxin 2-positive terminals do not show labeling for syntaxin 1 (arrowheads). Similarly, Most labeling for syntaxin 1 does not colocalize with labeling for syntaxin 2. Images shown from a projection of 13 optical sections with total thickness of 1.75 μm. **D–F: **Inspection at higher magnification shows that syntaxin 2 labeling is typically present in puncta that show little or no syntaxin 1 labeling, although a few puncta that show strong colocalization of labels are present (arrowheads). Colocalization associated with diffuse labeling, which is probably not associated with release sites, is also present. Images shown from a single optical plane. INL, inner nuclear layer, GCL, ganglion cell layer. Scale bars = 10 μm for A–C; 5 μm for D–F.

The localization of syntaxin 2 labeling to amacrine cells, suggested that syntaxin 2 potentially might have some redundancy with syntaxin 1. This was directly examined by double labeling for syntaxin 2 and syntaxin 1. Some colocalization of syntaxin 1 and syntaxin 2 labeling was observed in the IPL (Fig. 7A–C). Inspection by confocal microscopy at higher magnification, however, showed that syntaxin 2 labeling was largely independent of syntaxin 1 labeling (Fig. 7D–F), although discrete syntaxin 2-positive puncta sometimes did colocalize with diffuse labeling for syntaxin 1. The differences in subcellular distribution between syntaxin 1 and syntaxin 2 suggest that these two isoforms are non-redundant, even though they are co-expressed by amacrine cells.

**Figure 8:**
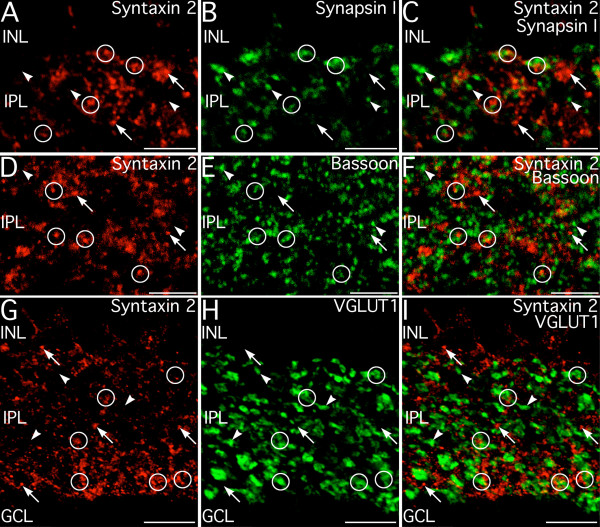
**Syntaxin 2 is primarily localized outside presynaptic terminals. A–C: **Syntaxin 2 is rarely localized to conventional presynaptic terminals in the inner plexiform layer (IPL). Syntaxin 2-positive puncta (arrows) do not colocalize with conventional synapses labeled for synapsin 1 (arrowheads). However, syntaxin 2-positive puncta are often closely apposed to synapsin I-positive presynaptic terminals (circles). Images shown from a projection of 9 optical sections with total thickness of 1.16 μm. **D–F: **Syntaxin 2 labeling (arrows) does not colocalize with labeling for the presynaptic active zone protein, bassoon (arrowheads). However, syntaxin 2-positive puncta are frequently apposed to bassoon-positive synaptic active zones. Images shown is a projection of 14 optical sections with total thickness of 1.89 μm. **G–I: **Syntaxin 2 is not expressed in bipolar cell terminals. Syntaxin 2 labeling (arrows) does not colocalize with labeling for VGLUT1 (arrowheads) in bipolar cell terminals. However, syntaxin 2-positive puncta are frequently closely apposed to VGLUT1-positive bipolar cell terminals. Images shown from a projection of 11 optical sections with total thickness of 1.45 μm. INL, inner nuclear layer, GCL, ganglion cell layer. Scale bars = 5 μm for A–C; 10 μm for D–I.

The subcellular localization of syntaxin 2 suggested that it might be located in synaptic terminals lacking syntaxin 1, or that it might not be localized to a presynaptic compartment. To clarify this issue, double labeling for syntaxin 2 and known presynaptic markers was performed (Fig. 8). There was little colocalization of labeling for syntaxin 2 and the conventional synapse marker synapsin I, although close apposition of labeled puncta was common (Fig. 8A–C). Likewise, double labeling for syntaxin 2 and the presynaptic active zone protein, bassoon, showed little colocalization but often showed apposition (Fig. 8D–F). Double labeling for syntaxin 2 and VGLUT1 confirmed that syntaxin 2 was not present in the ribbon synapses of bipolar cells, although puncta labeled for syntaxin 2 were often closely apposed to bipolar cell terminals (Fig. 8G–I). Double labeling for syntaxin 2 and synaptic vesicle protein 2 (SV2) using a pan-specific antibody that recognizes the SV2 isoforms at both ribbon and conventional synapses [23], also showed little evidence of colocalization with syntaxin 2 (not shown). Together, these results indicate that syntaxin 2 was only rarely expressed in presynaptic conventional terminals and was not expressed presynaptically at bipolar cell ribbon synapses.

Double labeling for syntaxin 2 and known ganglion cell and Müller glial cell markers showed no colocalization (microtubule-associated protein-1, MAP-1; glutamine synthetase, respectively. Not shown). These results, together with the results above, confirmed that syntaxin 2 labeling was restricted to amacrine cells and their processes in the IPL.

### Syntaxin 4 is expressed in postsynaptic compartments in the OPL and IPL

**Figure 9:**
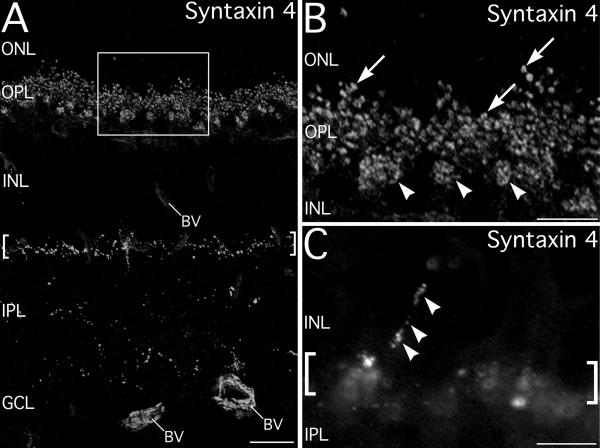
**Syntaxin 4 is highly restricted within both plexiform layers. A**. Low magnification image of syntaxin 4 labeling. Labeling in the outer plexiform layer (OPL) is present in two sets of puncta. One set of small individual puncta in the distal OPL and a second set of puncta found in larger clusters in the proximal OPL. In the inner plexiform layer (IPL), syntaxin 4 puncta form a prominent, narrow stratum (brackets) at the border with the inner nuclear layer (INL) and in sparsely distributed individual puncta deeper in the IPL. Labeling for syntaxin 4 also is present in blood vessels (BV). Box indicates area shown in panel B. **B: **High magnification image showing syntaxin 4 puncta in the OPL. Small puncta appear as singlets or doublets in the distal OPL (arrows). In the proximal OPL, syntaxin 4 labeling is found in large clusters of puncta (arrowheads). **C: **Conventional fluorescence image showing a series of syntaxin 4 puncta ascending through the INL (arrowheads). The stratum of syntaxin-4 positive puncta at the INL/IPL border is also visible (brackets). Panels shown in A and B are from a projection of 46 optical sections of 10.7 μm total thickness. ONL, outer nuclear layer; GCL, ganglion cell layer. Scale bars = 20 μm for A; 10 μm for B and C.

Labeling for syntaxin 4 was the most restricted of all the isoforms examined (Figs. 1 and 9). Syntaxin 4 labeling was present in numerous small puncta in the OPL (Fig. 9A). These puncta were distributed individually in the distal OPL or in clusters more proximally (Fig. 9B), similar to the distribution of rod and cone terminals. In the IPL, a thin stratum of syntaxin 4-positive puncta was present at the INL/IPL border, with a few other syntaxin 4-positive puncta scattered more proximally in the inner portion of the IPL (Fig. 9A). Syntaxin 4 also was present in strings of small puncta in the INL that resembled the processes of interplexiform cell processes coursing vertically toward the OPL, however, the precise origins of these processes is unknown (Fig. 9C). Syntaxin 4 labeling also was found in the walls of blood vessels, and was especially prominent in relatively large diameter vessels along the inner surface of the retina (Fig. 9A).

### Syntaxin 4 is present in horizontal cell processes post-synaptic to rod and cone terminals

**Figure 10:**
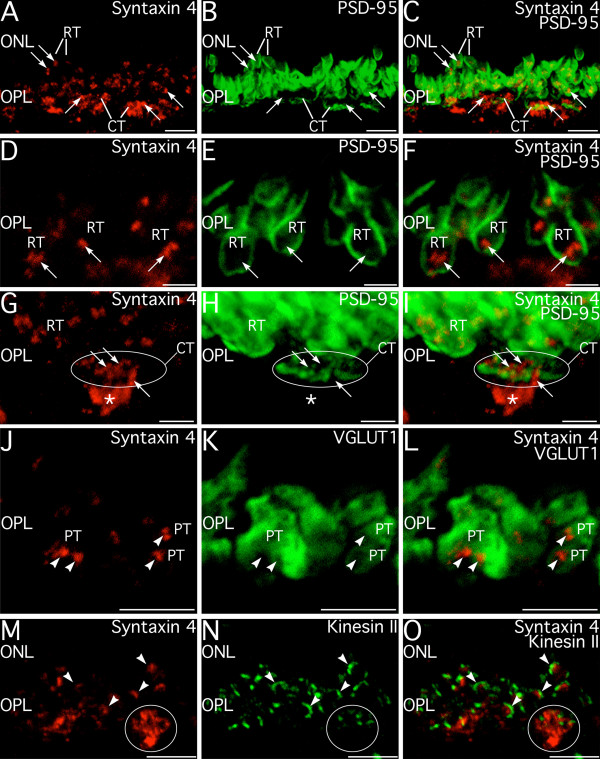
**Syntaxin 4 labeling in the outer plexiform layer (OPL) is associated with processes post-synaptic to the photoreceptor terminals, not the terminals themselves. A–C: **Double labeling for syntaxin 4 and the photoreceptor terminal marker PSD-95 show that syntaxin 4-positive puncta (arrows) are spatially associated with rod terminals (RT) in the distal OPL and with cone terminals (CT) in the proximal OPL. Images shown from a projection of 27 optical sections with total thickness of 3.79 μm. **D–F: **At high magnification, syntaxin 4-positive puncta in the distal OPL appear as singlets or doublets (arrows) enveloped by the terminals of rods (RT) labeled for PSD-95. Image shown is a projection of 22 optical sections with total thickness of 3.2 μm. **G–I: **In the proximal OPL, syntaxin 4 labeled structures (arrows) are associated with cone terminals (CT). Several syntaxin-4 positive puncta are enveloped by the cone terminal. In addition, syntaxin 4 labeling is localized to a cluster of processes in the OPL just proximal to the cone terminal (*) that can be seen to connect to the puncta within the cone terminal. Numerous rod terminals (RT) and associated syntaxin 4 puncta are visible distal to the cone terminal. Image shown is a projection of 34 optical sections with total thickness of 7.09 μm. **J–L: **Syntaxin 4-labeled structures (arrowheads) in the OPL do not colocalize with synaptic vesicles labeled for VGLUT1 in photoreceptor terminals (PT). Images shown from a projection of 13 optical sections with total thickness of 1.75 μm. **M–O: **Synaptic ribbons in rod and cone terminals do not label for syntaxin 4. However, syntaxin 4-positive puncta in the OPL are spatially associated with synaptic ribbons and often can be seen to follow the contours of the ribbons (arrowheads) labeled for kinesin II in rod and cone terminals. Circle indicates location of a cone terminal. Images shown from a projection of 12 optical sections with total thickness of 1.60 μm. ONL, outer nuclear layer. Scale bars = 5 μm for A–C, and J–O; 2 μm for D–I.

To determine the relationship of the syntaxin 4 positive puncta to photoreceptor terminals in the OPL, we examined double labeling for syntaxin 4 and markers associated with photoreceptor terminals (Fig. 10). Labeling for syntaxin 4 and post-synaptic density protein-95 KDa (PSD-95), which outlines the plasma membrane of rod and cone terminals [30], showed that small syntaxin 4 positive puncta in the distal OPL were associated with rod terminals and the larger syntaxin 4-positive clusters in the proximal OPL were associated with cone terminals (Fig. 10A–C). Examination at high magnification revealed that syntaxin 4 positive puncta were enveloped by rod and cone terminals and often appeared as a doublet or a crescent (Fig. 10D–F, respectively). Rod terminals enveloped one to four puncta; cone terminals enveloped numerous puncta. Patches of syntaxin-4 positive processes were often visible immediately below cone terminals and often connected to the puncta enveloped within the terminal (Fig. 10G–I), suggesting that syntaxin 4 labeling was associated with processes from second-order neurons. Labeling for syntaxin 4 and VGLUT1 were located adjacent to one another but did not colocalize (Fig. 10J–L), indicating that syntaxin 4 was not present within some highly localized pool of synaptic vesicles in the photoreceptor terminals. Syntaxin 4 labeling also did not colocalize with the synaptic ribbon markers kinesin 2 [31] or bassoon [17] (Fig. 10M–O; kinesin 2 shown), although the syntaxin 4-positive puncta were closely apposed to ribbons and often followed the contours of the ribbons. Together, these results indicate that syntaxin 4 is not present presynaptically in the rod or cone terminals, but is localized to processes of second-order neurons postsynaptic to the rod and cone terminals, including processes located within the ribbon synaptic complexes.

**Figure 11:**
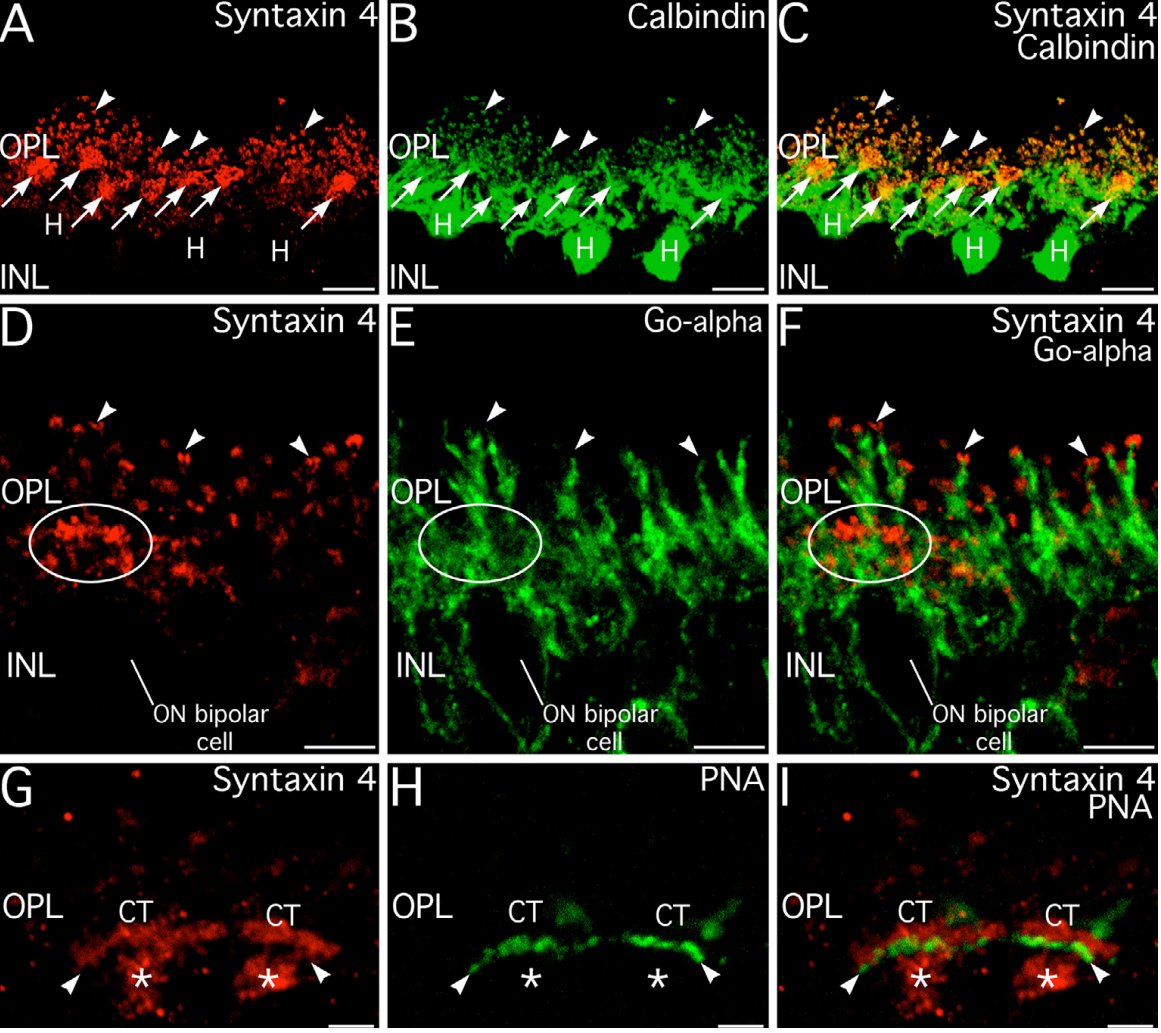
**Syntaxin 4 is localized to the tips of horizontal cell processes at ribbon synapses, but not to dendrites of ON- or OFF-bipolar cells. A–C: **Syntaxin 4 is present in the tips of horizontal cell processes in the ribbon synaptic complexes of rods and cones. Syntaxin 4 and calbindin, a horizontal cell marker, show double labeling in the individual puncta associated with rod terminals (arrowheads) and in the clusters of puncta associated with cone terminals (arrows) in the OPL, indicating that the syntaxin 4 is present at the tips of horizontal cell processes invading rod and cone terminals. Images shown from a projection of 37 optical sections with total thickness of 5.24 μm. **D–F: **Syntaxin 4 is not present in the dendrites of rod or ON-cone bipolar cells. Double labeling for syntaxin 4 and G_o_-alpha, a marker for rod and ON-cone bipolar cells, shows no colocalization. ON-bipolar cell dendrites reach up to the level of the syntaxin 4-positive puncta and terminate. In favorably oriented complexes in rod terminals (arrowheads), rod bipolar cell dendrites extending into the distal OPL terminate between the syntaxin 4-positive doublets corresponding to horizontal cell processes. No colocalization of syntaxin 4 and G_o_-alpha is present in ON-cone bipolar cell dendrites in cone terminals (ellipse). Image shown is a single optical section. **G–I: **Syntaxin 4 is not present at flat contacts between OFF-cone bipolar cells and cone terminals. Double labeling for syntaxin 4 and peanut agglutinin (PNA), a marker for flat contacts (arrowheads) between OFF-cone bipolar cells and cone terminals (CT) shows no colocalization. Syntaxin 4 labeling in processes directly proximal to the cone terminals is also visible (*). Images shown from a projection of 26 optical sections with total thickness of 6.71 μm. INL, inner nuclear layer. Scale bars = 10 μm for A–C; 5 μm for D–F; 2 μm for G–I.

A series of double labeling experiments combining labeling for syntaxin 4 with known markers for specific subsets of second-order neurons or their synapses was performed to identify precisely which second-order neurons expressed syntaxin 4 (Fig. 11). Double labeling for syntaxin 4 and calbindin, a marker for mouse horizontal cells [32], showed extensive colocalization of syntaxin 4 and calbindin labeling in the horizontal cell processes, directly demonstrating that syntaxin 4 was present in horizontal cell processes at the ribbon complexes of rods and cones (Fig. 11A–C). Double labeling for syntaxin 4 and G_o_α, a marker for ON-cone and rod bipolar cells and their dendrites [33], showed no colocalization, indicating that syntaxin 4 was absent from the dendrites of rod and ON-cone bipolar cells (Fig. 11D–F). The dendrites of the ON-cone and rod bipolar cells often were seen terminating just below and between syntaxin 4 positive puncta in horizontal cell processes. This arrangement precisely matches the ultrastructural arrangement of ON bipolar cell dendrites and horizontal cell processes in the ribbon synaptic complexes. Double labeling for syntaxin 4 and peanut agglutinin (PNA), which labels the flat contacts on the base of cone terminals, showed that syntaxin 4 was not present in the dendrites of OFF-cone bipolar cells where they form flat contacts onto the cone terminals (Fig. 11G–I). Together, these results indicate that syntaxin 4 is specifically localized to the post-synaptic processes of horizontal cells occupying the lateral position at the ribbon synaptic complex, but not in the dendrites of ON bipolar cells in the ribbon complexes or OFF-cone bipolar cell dendrites at flat contacts.

**Figure 12:**
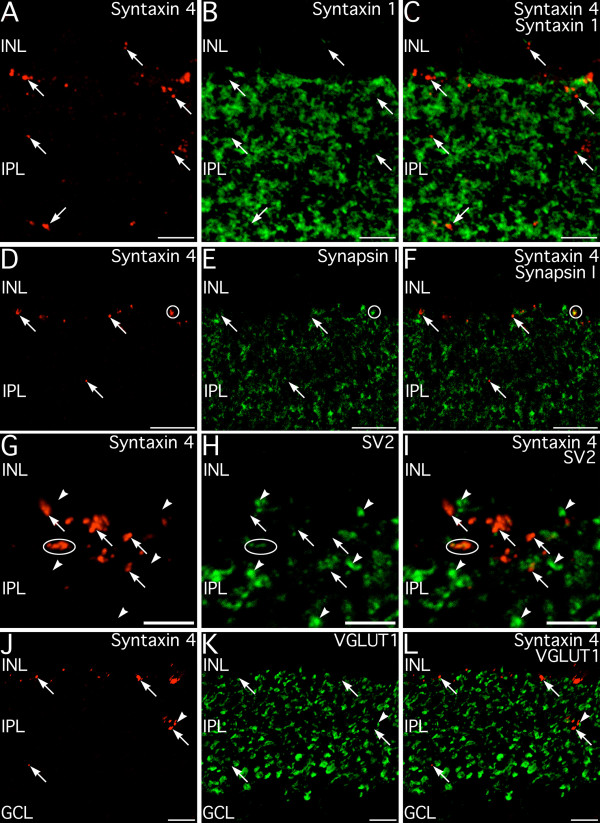
**Syntaxin 4 does not colocalize with syntaxin 1 or other presynaptic markers for conventional or ribbon synapses in the inner plexiform layer (IPL). A–C: **Syntaxin 4-positive puncta (arrows) found in the inner nuclear layer (INL) and IPL do not show labeling for syntaxin 1, which is expressed in the processes and conventional synapses of amacrine cells. Image shown is a projection of 15 optical sections with total thickness of 2.19 μm. **D–F: **Syntaxin 4 positive puncta (arrows) only very rarely show any colocalization with synapsin I (circle), a marker for conventional presynaptic terminals. Image shown is a projection of 10 optical sections with total thickness of 1.31 μm. **G–I: **Syntaxin 4-labeling (arrows) is only rarely associated with presynaptic terminals identified by labeling for the ubiquitous presynaptic marker synaptic vesicle protein 2 (SV2; arrowheads). Terminals showing weak colocalization are indicated by the ellipse. Image shown is a projection of 9 optical sections with total thickness of 1.17 μm. **J–L: **Syntaxin 4-positive puncta in the IPL (arrows) do not label for VGLUT1, indicating that syntaxin 4 is not present in bipolar cell terminals. Occasionally syntaxin 4-positive puncta make contact with VGLUT1-positive bipolar cell terminals (arrowhead). Image shown is a projection of 13 optical sections with total thickness of 1.78 μm. GCL, ganglion cell layer. Scale bars = 5 μm for A–C and G–I; 10 μm for D–F and J–L.

**Figure 13:**
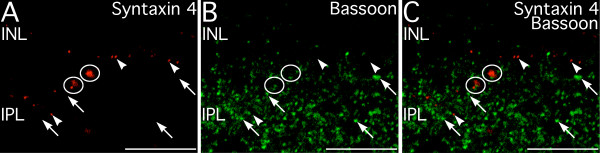
**Syntaxin 4 does not colocalize with the presynaptic active zone protein, bassoon, in the inner plexiform layer (IPL)**. **A–C: **Syntaxin 4-positive puncta in the IPL (arrowheads) do not show colocalization with presynaptic active zones containing bassoon (arrows). However, close apposition of syntaxin 4-positive puncta and bassoon-containing active zones is present (circles). Single confocal plane shown. Scale bars = 10 μm.

To better understand the potential functions of syntaxin 4 in the inner retina, double labeling for syntaxin 1, synapsin 1, SV2, and VGLUT1 was performed to characterize the relationship between the syntaxin 4-positive puncta and conventional and ribbon synaptic terminals in the IPL (Fig. 12). Labeling for syntaxin 4 only rarely showed any colocalization with any of these presynaptic markers in either the INL or IPL, however syntaxin 4-positive puncta were often apposed to conventional and ribbon synaptic terminals. Consistent with these findings, syntaxin 4 labeling was often apposed to active zones labeled for bassoon, but did not colocalize bassoon itself (Fig. 13). These results suggest syntaxin 4 in the IPL is located primarily in postsynaptic terminals.

### Syntaxin 4 puncta in the IPL interact with dopaminergic and GABAergic amacrine cells

**Figure 14:**
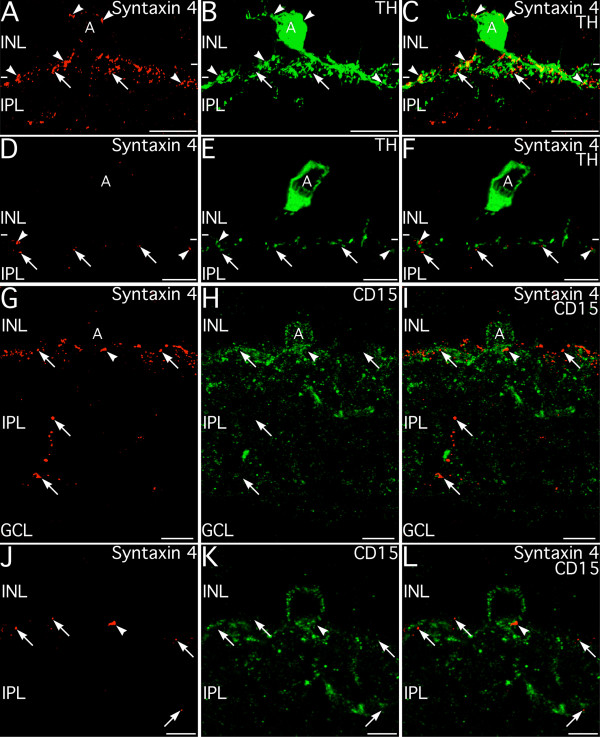
**Syntaxin 4-positive puncta in the distal inner plexiform layer (IPL) do not arise from dopaminergic or GABAergic CD15-positive amacrine cells**. **A–C: **Syntaxin 4-positive puncta form a plexus along the border of the inner nuclear layer (INL) and IPL that co-stratifies with the processes of dopaminergic amacrine cells (A) labeled for tyrosine hydroxylase (TH). Syntaxin 4 and TH labeling do not colocalize (see D–F), but syntaxin 4-positive puncta contact the processes and cell bodies of the dopaminergic cells (arrowheads). Images shown from a projection of 73 optical sections with total thickness of 10.5 μm. **D–F: **Higher magnification image of syntaxin 4-positive puncta (arrows and arrowheads) and TH-positive dopaminergic amacrine cell processes in the IPL in a single optical plane. There is no colocalization of labels indicating that the syntaxin 4-positive puncta do not arise from the dopaminergic amacrine cells, but there is often contact between the syntaxin 4-positive puncta and the TH-positive processes (arrowheads). **G–I: **Syntaxin 4-positive puncta (arrows) form a distinctive plexus that co-stratifies with processes from GABAergic CD15-positive amacrine cells (A). The processes of the CD15-positive amacrine cells do not label for syntaxin 4, but there is contact between syntaxin 4-positive puncta and the processes and cell body of CD15-positive amacrine cells (arrowhead). Images shown from a projection of 57 optical sections with total thickness of 8.16 μm. **J–L: **Higher magnification image of syntaxin 4 and CD15 double labeling visualized in a single optical plane. Syntaxin 4-positive puncta (arrows) co-stratify with the CD15-positive plexus in the IPL and often contact CD15-positive amacrine cells at the cell body or on processes (arrowhead). Images shown from a projection of 14 optical sections with total thickness of 1.89 μm. GCL, ganglion cell layer. Scale bars = 20 μm for A–C; 10 μm for D–I; 5 μm for J–L.

The plexus of syntaxin 4 puncta at the INL/IPL border is located precisely where dopaminergic amacrine cells and a population of GABAergic amacrine cells positive for CD15 have their plexes in the IPL [34,35], suggesting that the syntaxin 4-positive puncta in the IPL might arise from or interact with these cell types. Double labeling for syntaxin 4 and tyrosine hydroxylase (TH), a marker for the dopaminergic amacrine cells, showed that the syntaxin 4 puncta co-stratified with processes from dopaminergic amacrine cells (Fig. 14A–C). Close examination of the arrangement of TH and syntaxin 4 labeling showed that the syntaxin 4 labeling was not present in processes arising from the dopaminergic cells, although there was contact between the processes of the dopaminergic amacrine cells and the syntaxin 4 puncta (Fig. 14D–F). In addition, syntaxin 4-positive puncta in the INL were often apposed to the cell body and short processes that emerged from the dopaminergic cells into the INL (Fig. 14A–C). A similar arrangement between the processes and cell bodies of the CD15-positive cells and the syntaxin 4-positive puncta in the distal IPL was observed (Fig. 14G–L). These results suggest that the dopaminergic and CD15-positive amacrine cells ramifying in the distal IPL may provide input to the syntaxin 4-positive puncta.

**Figure 15:**
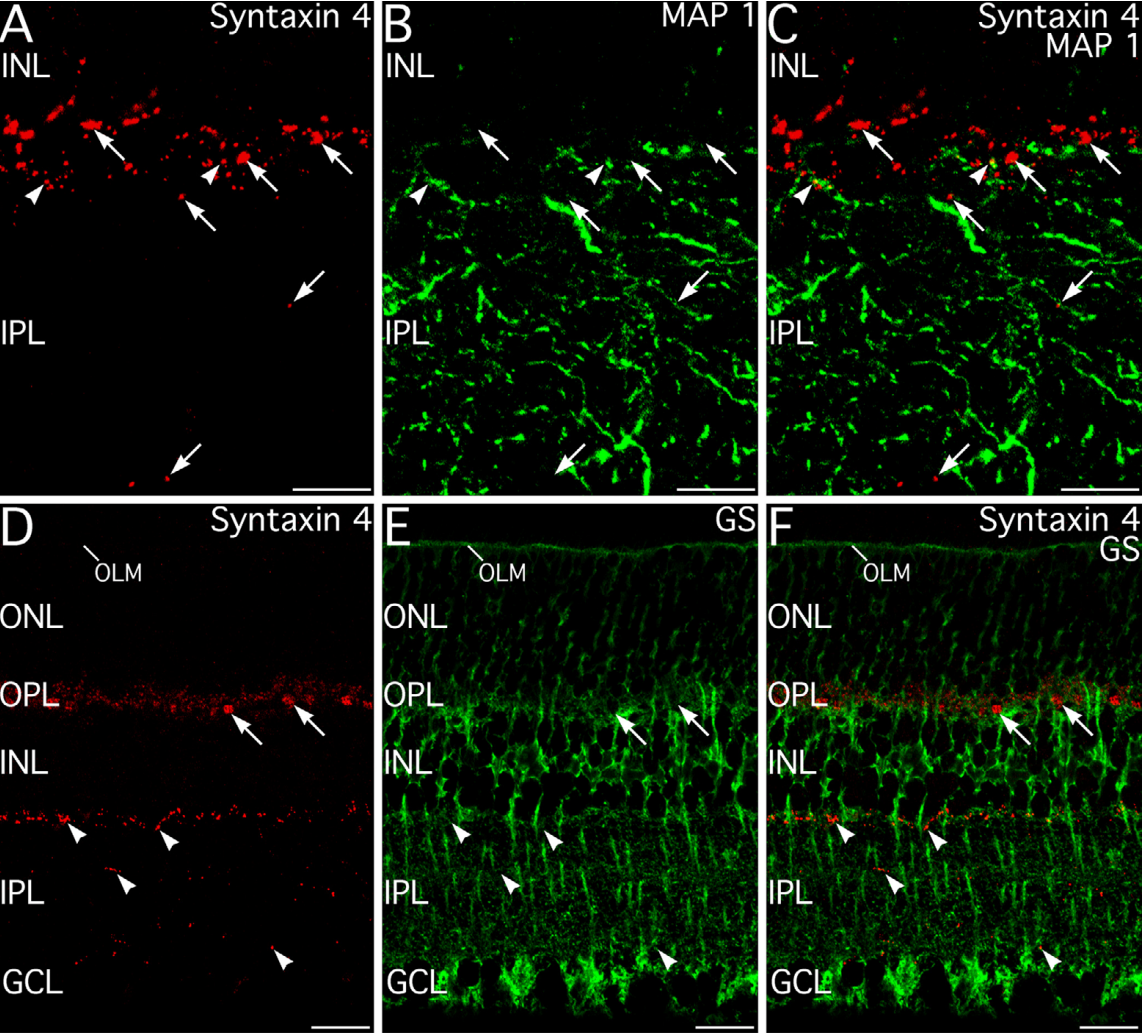
**Syntaxin 4 in the inner plexiform layer (IPL) is not associated with ganglion cell dendrites or with Müller glial cell processes. A–C: **Double labeling for syntaxin 4 and the ganglion cell dendrite marker, MAP 1. Syntaxin 4-positive puncta (arrows) form a plexus along the inner nuclear layer (INL)/IPL border with a few puncta present more proximally in the IPL. Ganglion cell dendrites labeled for MAP-1 are present throughout the thickness of the IPL, but do not show syntaxin 4 labeling. Contact between syntaxin 4-positive puncta and ganglion cell dendrites is seen only rarely (arrowheads). Images shown from a projection of 24 optical sections with total thickness of 3.35 μm. **D–F: **Double labeling for syntaxin 4 and glutamine synthetase (GS), a marker for Müller glial cells, shows no colocalization of syntaxin 4-positive puncta in the IPL (arrowheads) or in the outer plexiform layer (OPL; arrows). Images shown from a projection of 14 optical sections with total thickness of 3.35 μm. OLM, outer limiting membrane; ONL, outer nuclear layer; GCL, ganglion cell layer. Scale bars = 10 μm for A–C; 20 μm for D–F.

Double labeling for syntaxin 4 and the ganglion cell marker MAP-1 showed that syntaxin 4 labeling in the IPL was not associated with post-synaptic terminals of ganglion cell dendrites (Fig. 15A–C). Similarly, double labeling for syntaxin 4 and glutamine synthetase, a marker for Müller glial cells, showed no colocalization (Fig. 15D–F). Together with the results above, these findings suggest that the syntaxin 4-positive puncta in the INL and IPL do not arise from ganglion, bipolar or Müller cells, but are likely to arise from unidentified amacrine and/or interplexiform cells.

## Discussion

All four syntaxin isoforms associated with trafficking to the plasma membrane are expressed in the synaptic layers of the retina. Each isoform displays a unique distribution in the synaptic layers, with little colocalization of isoforms at the subcellular level, although they may be co-expressed within a cell. These findings strongly suggest that each syntaxin isoform mediates different trafficking events in the retina, and under normal conditions at least, serve different functions with little redundancy between isoforms. Syntaxins 1 and 3 are both presynaptic, but are found at different synapses. Syntaxins 2 and 4 generally do not appear to be presynaptic and are likely to have primary functions other than regulating neurotransmitter release.

### Differential distribution of trafficking proteins reflects functional differences

The distribution of trafficking proteins and their isoforms reflects functional differences. The distribution of several presynaptic proteins show distributions in the retina that differ according to the functional characteristics of synaptic release at conventional and ribbon synapses. Synapsins and rabphilin are present only in conventional retinal synapses [19,36]. Complexins III and IV and SV2B are found exclusively at ribbon synapses [20,23]. The expression of synaptotagmin isoforms, key vesicular Ca^++ ^sensors that regulate vesicle fusion [37], also can differ among conventional and ribbon synapses [16,38]. Content of presynaptic active zone cytomatrix proteins, such as piccolo and bassoon, also may not be uniform among synapses [[17,18], but also see [39]] and, at ribbon synapses at least, can be spatially segregated within an individual synapse [18,40]. Proteins associated with endocytosis, including dynamin, clathrin and amphiphysin, also are differentially distributed among ribbon and conventional synapses in a manner consistent with differences in the mode of endocytosis [41-46]. There is considerable diversity in post-synaptic trafficking as well, as post synaptic terminals exhibit a complex synapse-specific array of transmitter receptors and subunits, signaling, scaffolding and anchoring proteins [47-49]. Thus, the differential distribution of syntaxin isoforms observed here is likely to reflect functional differences in pre-synaptic, postsynaptic, and possibly, extrasynaptic compartments within the synaptic layers of the retina.

### Segregation and colocalization of syntaxin isoforms in the synaptic layers of the retina

There was no substantial colocalization of any syntaxin isoforms in either synaptic layer, although co-expression of syntaxin 1 and 2 were co-expressed in amacrine cells. Co-expression of multiple syntaxin isoforms that mediate trafficking to the plasma membrane is common, but the isoforms typically are spatially segregated to different subcellular compartments and mediate different trafficking functions [e.g., [7,8,50-52]; reviewed in [1,6]]. This is consistent with the current findings for syntaxin 1 and 2 which were co-expressed in amacrine cells, but differentially distributed at the subcellular level. Thus, each syntaxin isoform is likely to mediate a unique set of cell- and/or synapse-specific vesicular trafficking events in the synaptic layers of the retina, with little functional redundancy.

The large variety of syntaxin isoforms and their localization to specific subcellular compartments is thought to contribute to the specificity of vesicular trafficking to appropriate target membranes. Syntaxins can interact promiscuously with other SNAREs in vitro, but in vivo each syntaxin isoform has preferred binding partners [4,5,10,53]. Thus, the specific binding partners available to complex with syntaxin also may contribute to the regulation of membrane fusion and targeting. Presynaptic proteins that interact with syntaxins, including VAMPs and complexins, show synapse-specific distribution in the synaptic layers of the retina [20-22,54]. Munc-13, another protein that interacts with syntaxins, also may show differential distribution among retinal synapses, although this is controversial [40,55]. The distribution of these proteins do not precisely parallel the distribution of the various syntaxins, suggesting many possible combinations may exist that could shape the complex, synapse-specific characteristics of synaptic vesicle trafficking and exocytosis. For example, the distribution of complexin isoforms, small presynaptic proteins that bind to syntaxin and stabilize the fusion core complex [56], does not match syntaxin isoform distribution [[20,54], this report]. Complexins 1 and 2 are differentially distributed among amacrine cells, which co-express syntaxin 1 and 2. Complexins 1 and/or 2 are also found in horizontal cells, which express syntaxin 4. Complexins 3 and 4 are unique to ribbon synapses but are expressed in a cell-specific manner among the ribbon synapses of photoreceptors and bipolar cells, which all contain the same syntaxin, syntaxin 3.

### Isoform-specific functions of syntaxins 1 through 4 in the retina

The functional consequences of cellular and subcellular segregation of the various syntaxin isoforms in the plexiform layers of the retina are not yet known, as direct functional data are currently lacking. Potential trafficking functions that may be associated specifically with each syntaxin isoform in the retina are discussed below.

#### Syntaxins 1 and 3

The current study directly confirms that syntaxins 1 and 3 are the principal presynaptic syntaxins in the retina [[12]; this report] and extend previous findings by directly demonstrating that syntaxins 1 and 3 do not colocalize and that syntaxin 3 is present in all retinal ribbon synapses.

Our double-labeling studies confirm that the synaptic localization of syntaxin 1 is restricted to conventional synaptic terminals which show transient release characteristics and typically release an inhibitory amino acid transmitter, GABA or glycine, in the retina [15,29]. These results are consistent with previous reports in retina [12,23,36,57-59]. In contrast, syntaxin 3 was found exclusively at ribbon synapses of photoreceptors and bipolar cells, which are complex synapses organized around a lamellar synaptic ribbon and show very high, sustained rates of glutamate release, likely mediated by compound vesicle fusion [13,14]. This is consistent with previous results [12]. Syntaxin 3 may confer some specific advantage for rapid compound fusion of multiple vesicles for rapid transmitter release from photoreceptor terminals. Syntaxin 3 is known to localize to the membrane of secretory vesicles in the acinar cells of the pancreas and gastric parietal cells [8,60]. The current study also directly establishes that syntaxin 3 is not present at the putative glutamatergic conventional synapses of the VGLUT3 amacrine cells [24-26], indicating that syntaxin 3 is not specifically associated with glutamatergic transmission. Thus, syntaxins 1 and 3 segregate specifically according to the architectural and functional characteristics of the synapses.

Syntaxins 1 and 3 also were found extrasynaptically. Syntaxin 1 was diffusely distributed along amacrine cell processes, consistent with previous reports indicating that syntaxin 1 is not strictly localized to the synaptic active zone [e.g., [11,61]]. Extrasynaptic functions of syntaxin 1 in the retina are uncertain, but several possibilities exist. One possibility is a role in exocytosis of neuropeptides via dense cored vesicles, which can be released from any part of a neuron [62-65]. Such a role would be consistent with the well-known expression of a variety of neuropeptides by amacrine cells [66,67]. Another potential function for extrasynaptic syntaxin 1 is trafficking and regulation of transporters and channels. Syntaxin 1 associates specifically with a variety of neurotransmitter transporters [e.g., [68-74]] and ion channels [e.g., [75-77]]. Extrasynaptic syntaxins also have important roles in process growth and remodeling during neural development [78-81], and might have similar roles in process remodeling or plasticity associated with normal retinal function or pathology. Extrasynaptic pools of syntaxin 3 were present in photoreceptor inner segments and the cell bodies and axons of photoreceptors and bipolar cells. The function of syntaxin 3 in the photoreceptor inner segments is unclear, but an attractive candidate function is trafficking of outer segment proteins, such as opsins, which are trafficked via vesicles to the apical portion of the inner segment for assembly of outer segment discs [82-84]. Somatic pools of syntaxin 3 may be associated with standard "housekeeping" trafficking needs.

#### Syntaxin 2

Syntaxin 2 is expressed in amacrine cells and their processes in the IPL, similar to syntaxin 1. Syntaxin 2, however, does not colocalize with syntaxin 1, suggesting that syntaxins 1 and 2 are functionally complementary to one another despite being expressed by the same cells. Consistent with these findings, syntaxin 2 shows very little colocalization with conventional or ribbon presynaptic markers. These results indicate that syntaxin 2 must have principal functions other than presynaptic transmitter release despite its localization to the IPL.

One particularly attractive candidate function for syntaxin 2 in the IPL is trafficking of post-synaptic components, such as neurotransmitter receptors. Such a function would be consistent with the frequent apposition of syntaxin 2 to presynaptic terminals labeled for syntaxin 1 or syntaxin 3. However, this function has never been tested directly either in retina or in brain and the current study does not establish unequivocally whether syntaxin 2 is specifically localized to post-synaptic terminals. It is clear, however, that syntaxin 2 is not localized exclusively to postsynaptic terminals, as it is also found in the cell body and does not always align precisely with presynaptic markers. Thus, syntaxin 2 might serve extrasynaptic trafficking functions instead of, or in addition to, postsynaptic functions. A potential extrasynaptic function for syntaxin 2 is trafficking of proteins with neural functions that are not strictly localized to the synapse, such as transporters, ion channels or extrasynaptic transmitter receptors. Again, these functions have not been tested directly, but would be consistent with the colocalization of labeling for syntaxin 2 and GlyT1 in the IPL. Further studies to localize syntaxin 2 at the ultrastructural level would aid in resolving precisely which cellular compartments syntaxin 2 is present in within the processes of the amacrine cells.

Elsewhere in the body syntaxin 2 is known for mediating fusion of large secretory vesicles for exocytosis of proteins from non-neural cells [85-87]. By extension, syntaxin 2 might have a similar function in the IPL and mediate release of neuropeptides from amacrine cell processes via dense-cored vesicles as suggested above for syntaxin 1. Other important functions mediated by syntaxin 2 elsewhere in the body include cytokinesis [88] and the regulation of epithelial morphogenesis during development as a secreted, rather than an intracellular, protein [reviewed in [89,90]]. However, it seems unlikely that syntaxin 2 would have comparable functions in the adult retina.

#### Syntaxin 4

Syntaxin 4 had the most restricted distribution of all the isoforms studied, and is expressed specifically in horizontal cell processes at synaptic ribbon complexes in the terminals of rods and cones, and in small puncta in the IPL and INL. Syntaxin 4 also is found in non-neural cells associated with the retinal vasculature. The functions of syntaxin 4 in the retina have never been studied, but the distribution of syntaxin 4 did not overlap with the other syntaxin isoforms, strongly suggesting non-redundant functions. The lack of colocalization between syntaxin 4 and presynaptic markers for conventional and ribbon synapses indicate that the primary function of syntaxin 4 is not likely to be pre-synaptic transmitter release. On the other hand, syntaxin 4 was often found in puncta apposed to presynaptic markers, including synaptic ribbons and the active-zone protein bassoon, suggesting potential key functions in post-synaptic trafficking.

Syntaxin 4 in the OPL was restricted to the post-synaptic processes of horizontal cells, and was not found in bipolar cell dendrites or photoreceptor terminals. The presence of syntaxin 4 at the tips of horizontal cell processes in the ribbon synaptic complexes of rods and cones is intriguing. Photoreceptors provide glutamatergic input to horizontal cell processes flanking the synaptic ribbon via AMPA receptors [91,92]. The localization of syntaxin 4 to this site would be consistent with a role in local post-synaptic trafficking of neurotransmitter receptors and/or other signaling proteins to the horizontal cell plasma membrane, but this potentially important function has not been explored. Syntaxin 4 also might mediate trafficking of neurotransmitter transporters to the cell surface. Syntaxin 4 is critical to translocation of other transporter proteins, particularly glucose transporters, to the cell surface in response to receptor-mediated signals in non-neural cells [e.g., [93-95]] and also has been shown to mediate neurotransmitter transporter trafficking to the cell surface in cultured glioma cells [96].

Horizontal cell processes in the ribbon synaptic complex also provide inhibitory feedback to photoreceptors [[97]; reviewed in [98]]. The manner in which this feedback is provided is controversial. Horizontal cells have been suggested to provide this feedback by several different mechanisms: via the neurotransmitter GABA [99]; via electrical currents created by connexin hemi-channels [100,101], and by regulation of the pH in the synaptic cleft [98,102,103]. The presence of syntaxin 4 in the tips of horizontal cell processes could be interpreted as evidence for the existence of vesicular GABA release from adult horizontal cells. Support for this idea is provided by the expression of the vesicular GABA transporter and, sometimes, GAD and GABA in adult mammalian horizontal cells [104-107]. In addition, complexin 1 and/or 2, which interact specifically with syntaxins, are present in horizontal cells [54]. However, several key components needed for vesicular synaptic release of GABA, such as presynaptic active zones, SNAP-25, VAMP, and synaptotagmin family members have not been specifically identified in mature horizontal cell processes in the ribbon synaptic complex to date.

The current study showed only weak, diffuse labeling for syntaxin 1 in the OPL, consistent with previous reports [12,23,36,57-59]. In contrast, Hirano et al. [54] reported syntaxin 1 labeling in horizontal cell processes in the rabbit retina. This labeling may correspond to the weak syntaxin 1 labeling observed in the current and previous studies. The reasons for the relative differences in syntaxin 1 labeling intensity are not clear but may include species differences or subtle differences in labeling and visualization techniques. The high intensity of syntaxin 4 labeling relative to syntaxin 1 labeling in the OPL, however, suggests that syntaxin 4 is the predominant syntaxin for plasma membrane trafficking in horizontal cells. Resolution of the existence of GABAergic feedback from mammalian horizontal cells to photoreceptors and the potential roles of syntaxins 1 and 4 will require further investigation.

In the IPL, syntaxin 4 appears to be associated with processes from a small subset of amacrine and/or interplexiform cells, as double labeling showed no expression of syntaxin 4 in the processes of bipolar, ganglion or Müller cells. Most syntaxin 4 labeling in the IPL was concentrated in puncta at the INL/IPL border. These puncta do not arise from the dopaminergic or the CD15-positive GABAergic amacrine cells that stratify at this level of the IPL, but do appear to make contact with those cell types. The functional role of syntaxin 4 in the IPL is uncertain, but syntaxin 4 does not colocalize with presynaptic markers and is unlikely to have a major function in transmitter release. In contrast, syntaxin 4 was observed apposed to presynaptic active zones labeled for bassoon suggesting that syntaxin 4 in the IPL likely functions in postsynaptic trafficking or extrasynaptic transport functions.

## Conclusion

The synaptic layers of the retina show complex and diverse expression of syntaxin isoforms associated with trafficking to the plasma membrane. Each isoform shows a unique distribution in the synaptic layers, with little colocalization of isoforms at the cellular or subcellular level. These findings strongly suggest that each syntaxin isoform mediates different trafficking events in the synaptic layers of the retina and, under normal conditions at least, have little functional redundancy. Syntaxins 1 and 3 are both presynaptic, but are found at different synapses, Syntaxins 2 and 4 generally are not presynaptic and are likely to have principal functions in mediating post-synaptic and extrasynaptic trafficking rather than mediating neurotransmitter release.

## Methods

### Animals and tissue preparation

All studies were performed using the retina of adult mice (C57BL/6). Mice were kept on a 12 hour light:12 hour dark cycle. Food and water were available at all times. Light or dark-adapted mice were euthanized by rapid cervical dislocation, and the eyes were rapidly enucleated. The corneas were removed or punctured and the eyes were immersed immediately in 4% paraformaldehyde in 0.1 M cacodylate buffer (pH 7.4) for 15 minutes to overnight at 4°C. Best results were obtained using eyecups fixed for 15–30 minutes. Eyecups were rinsed in phosphate buffered saline (PBS, pH 7.4), cryoprotected in 30% sucrose in PBS, embedded in OCT mounting medium, and fast frozen in liquid nitrogen. Frozen sections (10–15 μm thickness) were collected onto gelatin-coated or electrostatically-charged slides and stored at -20°C until use. All animal procedures conformed to US Public Health Service and Institute for Laboratory Animal Research guidelines and were approved by the local Institutional Animal Care and Use Committee.

### Antibodies and antisera

Several monoclonal and polyclonal antibodies with well-characterized specificity for specific syntaxin isoforms were used in these studies. Anti-syntaxin 1 was a mouse monoclonal antibody (Sigma Chemical Company, St. Louis MO; diluted 1:500–1:1000) [57]. Anti-syntaxin 2 was a rabbit polyclonal antibody directed against the cytoplasmic domain of syntaxin 2 (Synaptic Systems, Göttingen Germany; diluted 1:100–1:200) [108]. Anti-syntaxin 3 was a rabbit polyclonal antibody directed against the cytoplasmic domain of syntaxin 3 (Novus Biologicals, Littleton, CO; diluted 1:750–1:1000) [5,109]. Anti-syntaxin 4 was a rabbit polyclonal antiserum directed against amino acids 2–23 of mouse syntaxin 4 (Chemicon International, Temecula, CA; diluted 1:100–1:200) [95,110]. Specificity of the syntaxin antibodies and antisera was confirmed by western blotting (see Fig. 1). Pre-adsorption of anti-syntaxin-4 with its peptide antigen eliminated labeling. An extensive panel of well-characterized cell- and synapse-specific marker antibodies and lectins was used for double labeling studies to further characterize syntaxin isoform distribution. Details of primary antisera and antibodies are provided in Table 1.

**Table 1:** Primary antibodies and lectins

**Antigen**	**Host**	**Dilution**	**Source (catalog #; clone #)**	**Reference**
Syntaxin 1	Mouse	1:500–1:1000	Sigma Chemical Company, St. Louis, MO (S0664; clone HPC-1)	57
Syntaxin 2	Rabbit	1:100–1:200	Synaptic Systems, Göttingen, Germany (110-022)	108
Syntaxin 3	Rabbit	1:750–1:1000	Novus Biologicals, Littleton, CO (AB4113)	5,109
Syntaxin 4	Rabbit	1:100–1:200	Chemicon International, Temecula, CA (AB5330)	95,110
Bassoon	Mouse	1:500	Stressgen Biotechnologies, Victoria, British Columbia, Canada (VAM-PS003; clone SAP7F407)	111
Calbindin	Mouse	1:300	Sigma Chemical Company, St. Louis, MO (C9848; clone CB955)	---
CD15	Mouse (IgM)	1:50	BD Biosciences Pharmingen, San Diego, CA (555400; cloneHI98)	35
G_o_α	Mouse	1:500–1:1,000	Chemicon International, Temecula, CA (MAB3073; clone 2A)	112
Glutamic Acid Decarboxylase, 65 kDa (GAD-65)	Mouse	1:500–1:1000	Chemicon International, Temecula, CA (MAB351; clone GAD-6)	113
Glycine transporter 1 (GlyT1)	Goat	1:2000	Chemicon International, Temecula, CA (AB1771)	---
Glutamine synthetase	Mouse	1:1000	Chemicon International, Temecula, CA (MAB302; clone GS6)	114
Kinesin II	Mouse	1:50	Covance Inc., Princeton, NJ (MMS198P; clone K2.4)	115
Microtubule Associated Protein 1 (MAP-1)	Mouse	1:300–1:500	Sigma Chemical Company, St. Louis, MO (M4278; clone HM-1)	116
Peanut Agglutinin (PNA)	---	1:10	Molecular Probes, Eugene OR	117
Postsynaptic density protein-95 Kda (PSD-95)	Mouse	1:100	Upstate Biotechnologies, Lake Placid, NY (05–494; clone K28/43)	---
Synaptic vesicle protein 2 (SV2)	Mouse	1:20–1:100	Dr. K Buckley, Harvard Medical School, Boston, MA	118
Synapsin I	Mouse	1:500–1:1000	Chemicon International, Temecula, CA (MAB355)	119
Tyrosine hydroxylase (TH)	Mouse	1:500	Chemicon International, Temecula, CA (MAB318; clone LNC1)	---
Vesicular glutamate transporter 1 (VGLUT1)	Guinea pig	1:2500–1:5000	Chemicon International, Temecula, CA (AB5905)	28
Vesicular glutamate transporter 3 (VGLUT3)	Guinea pig	1:2500	Chemicon International, Temecula, CA (AB5421)	26

Secondary antisera were raised in goat or sheep and were specific for IgGs from rabbit mouse, guinea pig or goat, or mouse IgM, according to the primary antibodies used. Secondary antisera were conjugated to Cy3 or Cy5 (Jackson Immunoresearch Laboratories, West Grove, PA) or AlexaFluor488 or AlexaFluor633 (Molecular Probes, Eugene, OR) and were used at a dilution of 1:200–1:500. All antibodies and lectins were diluted using "blocker" containing: 10% normal goat serum + 5% bovine serum albumin + 1% fish gelatin + 0.1–0.5% Triton X-100 in PBS (pH 7.4).

### Immunoblotting

Immunoblotting of mouse retinal and brain membrane homogenates was performed as described previously for analysis of retinal synaptic proteins [22,38]. Membrane homogenates of skeletal muscle and liver also were prepared as an additional positive control for syntaxin 4. Briefly, tissues were isolated and membrane homogenates were made by sonication and centrifugation. Proteins were then resolved by SDS-PAGE using 10% polyacrylamide gel and transferred to PVDF membranes, which were blocked, incubated in primary antibody, rinsed and incubated in secondary antibody. Labeled proteins were visualized using enhanced chemiluminescence.

### Immunolabeling and imaging

Immunolabeling and imaging of cryosections was performed as described previously [22,23,28]. Briefly, cryosections were thawed, treated with 1% NaBH_4 _to reduce autofluorescence, and treated with blocker to reduce non-specific labeling. Blocker was removed and primary antibody or, for double labeling experiments, a combination of primary antibodies from different hosts was applied for 2 days at 4°C. Cryosections were rinsed, then incubated in fluorescently labeled secondary antibody for 45 minutes to 1 hour at room temperature. After incubation with secondary antibody, sections were rinsed extensively, then coverslipped using Vectashield (Vector Laboratories, Burlingame, CA) or Prolong Gold (Molecular Probes, Eugene, OR) to retard bleaching of the fluorescent labels. Specificity of labeling methods was confirmed by omitting primary antibody or substituting normal rabbit serum for primary antibody. To assure that there was no bleedthrough of signals between fluorescence channels, specimens were labeled using only one primary antibody in combination with multiple secondary antibodies. These experiments revealed no bleedthrough between fluorescence channels. On the confocal microscope, bleedthrough between fluorescence channels was eliminated by adjusting laser power and detector sensitivity or by scanning channels sequentially.

Conventional greyscale fluorescence images were digitized from the microscope using frame averaging to reduce noise. Confocal microscopy was performed using a Leica TCS-SP2 confocal microscope (Leica Microsystems, Exton, PA). In all cases, image scale was calibrated, and brightness and contrast were adjusted if necessary to highlight specific labeling. To assess double labeling, matching images in the AlexaFluor488, Cy3, or Cy5/AlexaFluor633 channels were captured independently, pseudo-colored green, red, or blue and superimposed using Photoshop software (Adobe Systems, San Jose, CA).

## Authors' contributions

DMS performed immunolabeling and imaging and drafted the manuscript. RM performed studies and analysis of syntaxin 3. KMS helped design, conduct and interpret western blotting studies. BdP performed studies and analysis of syntaxin 2. All authors have approved the manuscript.
